# A Critical Review of Symptom Management Nursing Science on HIV-Related Fatigue and Sleep Disturbance

**DOI:** 10.3390/ijerph182010685

**Published:** 2021-10-12

**Authors:** Joachim G. Voss, Julie Barroso, Tongyao Wang

**Affiliations:** 1 Frances Payne Bolton School of Nursing, Case Western Reserve University, Cleveland, OH 44106, USA; jgv20@case.edu; 2 School of Nursing, Vanderbilt University, Nashville, TN 37240, USA; julie.v.barroso@vanderbilt.edu; 3 School of Nursing, Fudan University, Shanghai 200032, China

**Keywords:** fatigue, HIV, interventions, nursing, sleep disturbances, symptom management

## Abstract

Despite improved antiretroviral treatments, people living with HIV (PLWH) continue to suffer from HIV-related fatigue and sleep disturbances. We first reviewed the definition, etiology, and research breakthroughs of HIV-related fatigue and sleep disturbances, then analyzed nurse-led symptom management studies to describe their efficacy and make recommendations for future symptom research. We searched PubMed, CINAHL, PsycInfo, Psych and Behavioral Sciences Collection, and Scopus to identify nurse-led studies on symptom management for PLWH in the past 20 years. A total of 13 experimental or quasi-experimental studies were identified. The types of interventions included exercise, cognitive behavioral therapy, coaching, and symptom management manualized self-care activities. Currently, we cannot recommend with certainty any of the tested symptom management strategies to reduce fatigue or sleep disturbances. The current findings need to be confirmed and expanded to understand optimal dosing and sustainability.

## 1. Introduction

Symptom management is core to nursing science, and research on symptom management is a priority for people living with HIV (PLWH). Despite antiretroviral treatments being globally available, quality of life continues to remain poor for some PLWH due to their experiences of HIV-related symptoms. The term ‘HIV-related symptom’ has not been defined but has been used extensively in the nursing literature, which includes as many as 72 symptoms that were not problematic before the onset of HIV infection. We considered any new symptom that occurs after the initial infection to be an HIV-related symptom [1]. The prevalence rates of HIV-related fatigue and sleep remain high [2,3,4]; a national study (N = 1597) in 2017 showed that 65% of PLWH experienced worsening fatigue after the onset of HIV infection [5]. Meanwhile, more than half (56%) of PLWH reported daytime sleepiness or drowsiness from impaired quality of sleep as their second most distressing symptom from both the CNICS cohort (Centre for AIDS Research Network of Integrated Clinical System, N = 5370) [6] and Lee’s Symptom and Genetics Project (N = 317) [7]. 

Our review of the literature on symptom management of HIV-related fatigue and sleep disturbances was guided by the University of California San Francisco (UCSF) symptom management model. This middle range theoretical framework was developed to guide the symptom management research on three dimensions including symptom experience, symptom management strategy, and outcomes of symptom status [8]. For this review, we will briefly introduce each symptom with a definition, etiology, breakthroughs in research, and gaps in our understanding. This is followed by a review of the literature from the past 20 years on nurse-led symptom management studies of HIV-related fatigue and sleep disturbances, to highlight the unique contribution of nurse investigators.

### 1.1. What Is HIV-Related Fatigue?

Fatigue is the most prevalent symptom among PLWH [9,10], and is defined as a subjective, unpleasant, potentially disabling, chronic symptom characterized by physical and/or psychological exhaustion (adapted from [11]). Fatigue has an adverse impact on the continuum of care; people may be too tired to get their medications from a pharmacy or to take them once they do get them [12,13]. When comparing case and control patients in South Africa after ≥5 months of first-line antiretroviral therapy (ART), Marconi et al. found fatigue predicted virologic failure independent of adherence measures [14]. HIV-related fatigue is multi-causal and strongly associated with psychological causes, particularly stress and stressful life events [15,16]. More recent work from Zuniga and colleagues (2020) showed in a sample of N = 32 PLWH that elevation in adiponectin, serum amyloid A, and soluble interleukin-1 receptor type II levels were highly predictive of fatigue. This could indicate that the chronic inflammatory processes of low-grade HIV within the cardiovascular system and the brain play a major role in the presence of fatigue [17]. 

### 1.2. What Are HIV-Related Sleep Disturbances?

Sleep disturbance among PLWH is another frequently reported symptom [18], which is uniquely different from populations with other diseases in terms of patterns and severity. HIV-related sleep disturbance is defined as ‘a disruption in the amount and quality of sleep that impairs functioning’ [18]. Lee et al. found that difficulty falling asleep (sleep onset >30min) and sleep fragmentation (wake after sleep onset >15% of the night) were the most common types of sleep disturbances in PLWH [19]. In the past 20 years, HIV-related sleep disturbances have slowly gained more attention due to their association with worsening of HIV-related symptom burden [20,21,22], disabling effects on overall health, and even failure to secure a job [23]. The etiology of HIV-related sleep disturbance remains poorly understood; however, sleep disturbances are a common side effect of many antiretroviral medications [16,24,25], and an Australian study (N = 522) found sleep disturbance was the most commonly reported ART side effect [26]. 

### 1.3. Breakthroughs on HIV-Related Fatigue and Sleep Disturbances Research

Breakthroughs in nursing science on understanding the unique etiology and pattern of each HIV symptom accelerated the progress of understanding fatigue and sleep disturbances and, thus, has the potential to improve focused interventions. One breakthrough for fatigue was the discovery by Barroso et al. [27] that HIV-related fatigue is chronic and persists for years, tending to remain at the same level of intensity and impact on functioning for years. In a longitudinal five-year study of 128 PLWH, fatigue did not spontaneously remit, nor was there a response shift on the measurement tools, but the level of fatigue and its impact on impairment of functioning (ability to accomplish activities of daily living (ADLs) and independent ADLs) remained essentially unchanged over a three-year period. Those who experienced low, medium, or high intensity fatigue levels remained in the same intensity level, respectively, throughout the three years of data collection. This clearly documents the chronicity of fatigue and the urgency to find interventions to help PLWH. 

A breakthrough in the treatment of sleep disturbances in PLWH was the discovery by Lee et al. [19]; in their cross-sectional design with 290 participants, Lee et al. found that the self-reported sleep quality was not related to quality of sleep objectively measured by wrist actigraphy and their overall HIV-associated symptom experiences. They advocated for greater effort in understanding patients’ specific sleep problems and designing interventions accordingly [19]. Even a person with a mild or moderate symptom experience could suffer tremendously and experience a high level of distress over the symptom.

### 1.4. Gaps in Our Understanding

We are currently unable to account for the lack of impact of more recent changes in HIV treatment, such as the ‘test and treat’ approaches or the single formulation of the triple combination therapy, which clearly improved adherence to medications but did not improve symptoms. The current intervention literature does not allow for assessment of whether changes in antiretroviral treatment changed the degree of symptom severity, duration, or impact, according to treatment modalities. Although the advent of and access to combination ART has drastically reduced morbidity and mortality rates, PLWH continue to suffer from multiple chronic HIV-related symptoms [28].

We have no way of distinguishing if the onset of new symptoms can be ascribed to the presence of the HIV infection alone or other issues such as the aging processes, developing comorbidities, or any other physical and psychological changes that may occur along their illness trajectory. At this point, we cannot say with certainty what drives the persistence of a particular symptom or multiple symptoms after the initial onset of HIV infection. We know, however, from longitudinal data that fatigue and sleep disturbances will persist for years and will not improve spontaneously without any meaningful interventions [29,30,31]. The literature is very clear that with the onset of HIV infection, PLWH begin and continue to experience fatigue and sleep disturbances. All these symptoms may be interrelated and have approached or exceeded prevalence rates of 50% in various samples of PLWH [5,32,33,34,35,36]. Symptom management has been a long-term goal of HIV nursing care through the examination of biological, social, and behavioral underpinnings of symptoms. Cognitive behavioral therapy for insomnia (CBT-I) is the first-line therapy recommended by the American Academy of Sleep Medicine for insomnia [37]. However, the effectiveness of other behavioral interventions warrants more evidence [38]. Due to the lack of symptom management strategies without medications, the authors focused on the studies that tested behavioral and biological interventions led by nurse investigators to ameliorate one or both symptoms in order to understand their effectiveness and long-term impact.

## 2. Materials and Methods 

**Figure 1:**
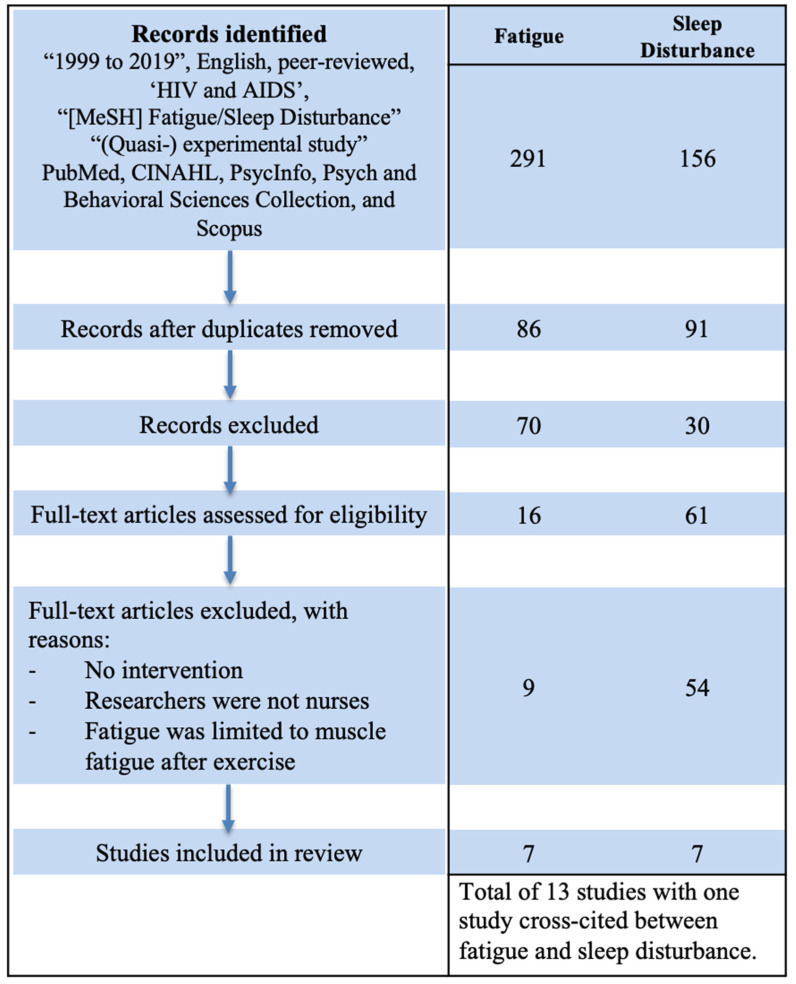
PRISMA Flowchart.

We searched for each symptom separately in five databases—PubMed, CINAHL, PsycInfo, Psych and Behavioral Sciences Collection, and Scopus—to identify nurse-led studies on symptom management for PLWH in the past 20 years (Figure 1). Our search criteria included: articles published between 1999–2019; written in English; peer reviewed; experimental or quasi-experimental studies conducted with adults (ages 18 years and older); and conducted by nurse investigators. The terms ‘HIV and AIDS’ AND ‘fatigue OR sleep disturbances’ were included as keywords. Specific to fatigue, we excluded exercise interventions in which the measure of fatigue was the point at which physiological muscle fatigue was reached during exercise, since this is not an HIV-related symptom.

## 3. Results

A total of 13 studies met our search criteria and were identified: one study on both fatigue and sleep disturbances symptoms, six additional studies on fatigue only, and six on sleep disturbances only (Figure 1—PRISMA Table). All studies were experimental, including nine RCTs and four quasi-experimental studies. Analytic methods used to examine the impact of the study interventions between baseline and post-intervention or between control and intervention groups included Wilcoxon signed-rank tests, multiple analysis of variance (MANOVA), analysis of covariance (ANCOVA) models, paired t-tests, and multilevel regression models (MLM). Studies were identified and categorized based on the three components of UCSF symptom management model: (1) symptom experience, (2) symptom management strategy, and (3) outcomes of symptom status in Table 1 and Table 2.

Symptom experience refers to the study participants’ experiences before the intervention. The symptom management strategy depicts the components of each study intervention. Outcome of symptom status was data collected post-intervention for evaluating the symptom management strategy’s effectiveness.

### 3.1. Fatigue Management Strategies

Fatigue management studies (N = 7) took diverse approaches, including exercise, cognitive behavioral therapy (CBT), the use of symptom management guidelines, and treating a co-morbid symptom with the goal of reducing fatigue. There were two studies which examined exercise programs to reduce HIV-related fatigue [39,40], and two studies that examined CBT, delivered in person [41] or via an app [42]. One study examined the use of symptom management guidelines with hospitalized patients living with HIV and fatigue [43]; one study examined the treatment of depression to determine the effects of reducing depression on fatigue [44]; and one study examined the treatment of sleep disturbances to determine the effect of reducing sleep disturbances on fatigue [45]. The Zhu et al. [43] study was conducted in China; the other six were conducted in the US. Four studies were randomized controlled trials (RCTs), two studies were pilot RCTs, and one was a pre-/post quasi-experimental design. Sample sizes ranged from 30 to 234. In those studies that reported this information, intervention doses varied widely, from three times/week for 12 weeks to treatments at two, four, and six weeks. Data were collected at baseline and then at widely varying intervals, with one study collecting data for a year, while most stopped data collection after three months or less post-intervention. Two studies used the HIV-Related Fatigue Scale, and the other five studies each used different fatigue measures, making comparison of results difficult. 

In a randomized controlled trial, Barroso et al. (2016) analyzed data from a sample of people living with HIV who were randomized to receive enhanced usual care for depression, or a depression treatment model called measurement-based care (MBC). Participants (n = 234) in this depression treatment trial who experienced a stronger depression response (greater improvement in depression scores) had larger decreases in fatigue. However, even among those who demonstrated a full depression response, nearly three-quarters continued to have either moderate or severe fatigue, supporting the belief that these are two separate constructs and must be treated as such. The treatment group experienced improvements in depression.

We also found two exercise interventions for fatigue management. The Jaggers et al. study implemented a supervised exercise program of aerobic and resistance training [40], and while there was a decrease on the POMS (Profile of Mood States) fatigue sub-scale from pre- to post-intervention, it was not statistically significant. Goulding et al. [39] enrolled a sample of older people to complete 12 weeks of moderate intensity exercise, then randomized them to complete another 12 weeks of moderate or high intensity exercise [39]. High intensity exercise was associated with greater improvements in vitality/fatigue in weeks 13–24 compared to moderate intensity. 

In the area of mind/body interventions, CBT also showed potential to improve fatigue. Doerfler and Goodfellow [41] found that individual CBT when compared to usual care was effective in reducing fatigue in PLWH on ART; however, the intervention did not have a sustained effect at the 90-day measurement. In the second study using CBT, Barroso et al. [42] developed an app based on cognitive behavioral stress management (CBSM). At three months, findings showed an improvement in fatigue, with completers (those who completed at least 80% of the intervention modules) having a sustained, significant reduction in fatigue intensity and impairment of fatigue-related functioning. 

Another two studies used behavioral-educational strategies to manage fatigue. Zhu et al. (2018) implemented an HIV symptom management guideline in an inpatient unit in Shanghai, China, with fatigue being one of the targeted symptoms. Frequency of fatigue was lower in the intervention group but was not statistically significant [43]. Finally, in a randomized controlled pilot study, Lee, Jong, and Gay [45] tested a behavioral-educational intervention to reduce fatigue through education about daytime behaviors and nighttime sleep behaviors. Participants were living with HIV, between 45–75 years old, unemployed, and fatigued. At the conclusion of the study, the intervention group had significantly improved fatigue severity scores and symptom burden over time, especially in the frequency of fatigue.

### 3.2. Sleep Disturbance Management Strategies

Seven studies, examining six interventions on sleep disturbances, met our inclusion criteria. Two of the seven studies analyzed the ‘Sleep B.E.T.T.E.R’ intervention, first conducting an efficacy trial [46] and then an RCT [45]. Overall, nurse-led interventions to address HIV-related sleep disturbances were composed of two mechanisms targeting the biological response or sleep hygiene behaviors. Three biological response studies included acupuncture treatment [47], 30-day caffeine withdrawal [48], and transcranial direct current stimulation (tDCS) [49]. The other three psychoeducational intervention studies on sleep hygiene included SystemCHANGETM-HIV intervention [50], Brief Behavioral Treatment for Insomnia [51], and ‘Sleep B.E.T.T.E.R’ [45]. The total number of participants ranged from 12 to 120 across all studies and the studies took place between 2001 and 2019. One study, ‘Sleep B.E.T.T.E.R.’, included only women living with HIV and the others included all genders. Of note, the ‘Sleep B.E.T.T.E.R.’ intervention was tested in two separate studies [45,46], one with women-only participants, and the other with all gender participants; nevertheless, both studies demonstrated significant improvement in sleep quality. The length of the interventions ranged from 4 to 10 weeks with varied frequency and doses of intervention over time.

Three interventions were developed primarily to target the biological components of sleep quality. In 2001, the first HIV-related sleep disturbance quasi-experimental study in our search was conducted by a nurse and a professional acupuncturist. They recruited 21 participants and provided five weeks of individualized acupuncture treatment [47]. Not only did they find a 32% improvement in sleep quality, they also learned that as the acupuncturist developed a personal treatment plan for each individual, pain was the most common patient-reported cause for poor sleep [47]. Improvements in length of sleep (*p* = 0.05) and total minutes of awakening (*p* = 0.05) were statistically significant, but no change was found in the amount of time that people needed to fall asleep, also called sleep latency (*p* = 0.87) [47]. In 2003, Dreher conducted an RCT to test the effects of a 30-day gradual caffeine withdrawal on sleep quality in 120 PLWH. Participants with 90% caffeine reduction experienced a 35% significant improvement in sleep quality compared with participants who had a 6% caffeine reduction [48]. In 2019, Cody et al. tested another two biological interventions, using speed of processing training (SOPs, an interactive computerized exercise to improve speed and accuracy to visual stimuli) or transcranial direct current stimulation (tDCS) over five weeks (1 h twice a week) in older adults (ages > 50) living with HIV, and neither of these interventions improved sleep quality [49]. tDCS is defined as a non-invasive procedure to slightly change the membrane potential of neurons with a static, direct electrical current to stimulate the brain [52].

An additional four intervention studies tested used educational and coaching sessions to promote sleep hygiene behaviors and therefore improve quality of sleep. In 2013, the ‘SystemCHANGETM-HIV intervention’ was tested; it was comprised of ten weekly sessions on different topics of HIV management, including sleep hygiene and behavioral modification strategies based on the SystemCHANGE theory. The theory encourages small environmental or behavioral changes with a goal of improving overall health [50]. The randomized controlled trial recruited 40 participants but showed no significant effect in sleep outcomes [50]. In 2018, Buchanan et al. developed the intervention called the Brief Behavioral Treatment for Insomnia (BBTI) based on three principles, including sleep restriction, stimulus control, and circadian mechanism. The interventionist worked with each participant to practice these principles and provided sleep hygiene education as well. The BBTI was the first intervention study to primarily facilitate behavioral change by working with everyone to agree on a set schedule of sleeping and rising. In the feasibility test of the intervention in 12 clinically diagnosed insomnia patients, intervention participants demonstrated fewer symptoms of insomnia and had a statistically significant increase in clinical sleep outcomes [51]. Furthermore, they also found that the BBTI was well accepted and rated favorably by PLWH. Another intervention, the ‘Sleep B.E.T.T.E.R’ (Bedroom, Exercise, Tension, Time to sleep, Eating, drinking and drugs, Rhythm) program, was designed in 2008 to address sleep disturbance issues by providing a 30-minute instructional session on sleep hygiene and advising participants to practice sleep hygiene in the following week (N = 30 female participants only); the post-intervention actigraphy results showed a significant reduction in sleep disturbance only, and minimal improvement in overall sleep [46]. Later in 2019, Lee and her colleagues extended the ‘Sleep B.E.T.T.E.R’ intervention to 60 min by providing additional sleep hygiene devices, such as a white noise fan, eye mask, or caffeine-free tea, and adding weekly booster sessions over four weeks [45]. When the modified intervention was tested in an RCT (N = 55), participants experienced significantly improved sleep quality.

## 4. Discussion

### 4.1. Fatigue

With only seven intervention studies focused on fatigue severity using three different interventions, there are clearly gaps in the evidence. We found that CBT interventions were a promising approach to help PLWH dealing with their experiences of fatigue. However, we are not at the level to recommend CBT as an evidence-based practice, for several reasons. In Doerfler and colleagues’ (2016) study, 50% of participants did not complete the study (we do not know the reason for this attrition), and the differences first observed after 60 days were not sustained at 90 days. One explanation could be that participants went back to their old behaviors and lost the newly learned effects. The loss of participants and the sustained effect was much better in the pilot study by Barroso and colleagues (2020). This could have happened for two reasons: the intervention involved ten sessions, and the intervention delivery mode changed from in-person to a mobile app delivery. Accessibility and convenience might be major drivers in acceptability of behavioral interventions.

Jaggers and colleagues (2015), combining aerobic-resistance exercises for their intervention, also lost more than 50% of participants by the end of the 12-week study. The findings from that study did not show improvement in fatigue but significant changes in depression scores, which could be for several reasons: six weeks of two exercise sessions might not be enough of an exercise dose to cause significant changes in fatigue, depressive symptoms might improve faster than fatigue, or fatigue interventions need to be longer before the positive effects can be perceived. The results of Goulding and colleagues’ (2019) exercise intervention would support the hypothesis that the dosing of the exercise intervention needs to be longer and increased in intensity. In their 24-week-long study, they found that fatigue improved in those on a high intensity exercise regimen versus the group in the moderate intensity regimen.

A close relationship between depression and fatigue was demonstrated in Barroso and colleagues’ study in 2016, where treatment for depression recommended by a clinically supervised care manager to the HIV provider made a significant difference in fatigue. While highly promising, we are not at the point to make an evidence-based recommendation about this new care practice. However, these findings clearly demonstrate that depression and fatigue should always be screened together, and it seems that depressive symptoms are more responsive to shorter interventions than fatigue is. The final study by Zhu and colleagues (2019), consisting of a symptom management module only, showed non-significant decreases in fatigue and depression scores, but we do not know anything about the intervention itself, the frequency of the intervention, the manual of the intervention, or the activities that PLWH were supposed to have practiced during the study period.

### 4.2. Sleep Disturbances 

With seven studies focused on sleep disturbances related to HIV, the two oldest studies from 2001 and 2003 used acupuncture and caffeine withdrawal, and both improved sleep quality. The caffeine study points to a clear dose effect as people who reduced caffeine the most experienced the greatest benefit [48]. We have no information about the long-term impact of either intervention, and both were conducted long before we changed the triple combination drugs into a single tablet or moved to a test and treat modality. Caffeine withdrawal and acupuncture as interventions to improve sleep disturbances have not been repeated in any nurse-led study. The other four studies were focused on behavioral interventions through either four CBT sessions, 10 different behavior modification sessions focused on sleep modifications, or a single sleep intervention with personal goal setting that increased from 30–60 min. The findings would clearly point towards a dose effect with a higher dose being more effective, to allow the participants to integrate this new behavior in their life and to form new routines around it, but more research is needed about the content of the intervention and its delivery. The studies with a single session need to establish long-term effects for us to recommend them as an innovative practice for clinicians.

A major challenge in management of sleep disturbances is that these symptoms are multifactorial, making it difficult for a single intervention to address all factors. Furthermore, how much participants are bothered by sleep disturbances is independent of the total number of hours of sleep [19], which indicates that other individual and environmental factors contribute to the variations in sleep quality. For example, the group with difficulties falling asleep experienced the worst symptom burden, while the group with high levels of frequent awakenings had a similar level of symptom experience as the good sleep group [19]. Psychological strains induced by the life-changing HIV diagnosis such as HIV-related stigma, fear, and lack of social support, especially for women living with HIV, have not been considered when addressing poor sleep quality [53,54] and require more study.

We found interventions targeting either biological pathways or sleep hygiene behaviors. Although biological interventions are effective, disadvantages like an inability to address other sleep disturbance factors (environmental and safety), the high cost of delivering in-person interventions (CBT), and a lack of sustainable effect after the intervention (30-minute sleep counseling and goal setting), need to be better understood. While educational behavioral interventions were well accepted and favorably received by PLWH, the feasibility and efficacy of the educational interventions still require further study. Overall, studies exploring the mechanisms of HIV-related sleep disturbance remain few and far between.

### 4.3. Future Research Implications

Where does this leave us in terms of recommendations for the future? We need more data on these interventions, and larger studies that are fully powered to determine their short- and long-term effects, particularly those with a focus on exercise and those with a cognitive behavioral approach. Most studies had very small sample sizes, and three were feasibility studies. However, it is a start. Researchers who want to intervene to reduce HIV-related fatigue and sleep disturbances need to consider that: (1) CBT and tailored behavioral educational interventions showed the most promise/efficacy; (2) higher doses of the intervention demonstrated better results and for a longer period; and (3) none of the studies tested the need for a booster of the intervention.

In terms of effectiveness, we need to conduct studies that help us look at whether there is a sustained response to an intervention (another place in which wearable technology would help us). If the long-term effect fades, what type of booster and what mode of delivery are needed to be most effective? Regarding the nature of intervention studies, future work needs to clarify the appropriate dosing of an intervention, and account for the differing responses to an intervention based on gender, age, and ethnic differences, to name a few. If there are multiple components to an intervention, we need to distill that intervention down to its critical essence and test this across various groups of PLWH. 

We still treat fatigue and sleep disturbances as if they are the same phenotype for any person who experiences them. But our colleagues in cancer have shown that the opposite is the case [55]. The HIV nursing research community is currently challenged by not knowing whether phenotypic differences among individuals impact the intervention dosing, and how we should use that knowledge in intervention studies. There are many areas open for future research, including whether phenotypic differences make it more difficult or easier to diagnosis a symptom, what the consequences might be if you have one or the other phenotype of a particular symptom, or what the long-term consequences of any lingering symptom might be in relation to disability. How would understanding of phenotype help us to decide who should receive a particular intervention first?

Despite many questions regarding racial, age, or sexual disparities, we do not have any guidance for those subgroups. Questions focused on access to care, access to therapists, availability of transportation, and level of disability or employment can be grouped into environmental factors that may have significant relationships to the development and persistence of HIV-related symptoms, but none of these have been explored and translated into meaningful interventions. Finally, an area largely unexplored area remains regarding which biomarkers could be useful to appropriately diagnose fatigue and that could demonstrate the effectiveness of behavioral interventions.

### 4.4. Future Clinical Implications

There are several general considerations for future investigation that are applicable across both symptoms. In terms of technological advances, we need to better understand the full capability of currently accessible tools (apps and wearable devices) to measure symptoms to provide treatment for them (e.g., measuring sleep quality, level of physical activity, heart rate variability). For patients who are in rural areas, further consideration of remote delivery of interventions needs to be considered, although considerable advances have occurred with mobile technology [56,57]. Transportation has long been cited as a barrier to accessing in-person treatment [58] and with the COVID-19 epidemic it has become clear that telemedicine and remote delivery of care are attainable without in-person visits.

Regarding policy implications, we need to identify stakeholders and healthcare systems that are involved in optimizing structural symptom management, for example, implementing sidewalks in cities for walking interventions, building noise-cancelling walls along busy roads to reduce nightly disturbances, and increasing safety in neighborhoods so people can be active outside in the community. Researchers need to build a business case to policy makers and insurance companies about why investments in symptom management have a great return. Researchers also need to advocate for more funding for this work at a difficult time when our national economy is unstable and there are many competing health concerns, such as COVID-19.

There are contextual factors to consider as well. No illness is experienced in a vacuum, and neither are its symptoms; they all occur in some context. What is the combined effect of environmental factors on symptoms? We know little about how contextual factors such as weather conditions, access to sidewalks, or neighborhood safety impact an individual’s ability to deal with their symptoms and self-care activities. We also know little about how social determinants of health are tied to symptoms and their management. We need to know more about whether and what types of medications are being used, both over the counter and prescribed to treat a symptom, and their long-term consequences. Soporifics, stimulants, and antidepressants may have positive effects on a symptom short-term, but long-term use in a population with a high rate of substance abuse may result in more negative outcomes than we anticipated. Therefore, despite our best intentions, healthcare providers may be making the problem worse. Finally, Western healthcare providers have largely ignored the potential benefit of complementary and alternative therapies for a multitude of reasons, such as frequent clinic visits for acupuncture, the time to learn Tai Chi exercise, or finding instructions for guided imagery. Notably, with expanded multi-omics abilities, those complex interventions could now be explored with methods that account for their complexity and provide valuable answers to mechanisms, dosing, duration, and sustainability of effects. While rarely mentioned in the studies cited herein, they may have a major role to play due to fewer side effects, lower risk profiles, and lower costs.

## 5. Conclusions

HIV-related fatigue and sleep disturbances have been treated with exercise-related, social-behavioral, and biological interventions. The social-behavioral interventions, even with their limited numbers, have been found to have both long- and short-term effects. Focused symptom management research is needed to help those living with HIV-related symptoms so that we can prevent the high rates of PLWH who are classified with a disability and who do not return to an active work life. Policy makers should be encouraged to direct funding toward providing services that will help those living with HIV-related symptoms stay in their homes, maintain employment, and continue their journeys toward health and successful aging. Technology will need to play an integral role in the development of such interventions, as many people cannot get to a brick-and-mortar university or clinic to participate in intervention studies due to a lack of financial resources and/or access to transportation. While there are no data to help us understand if there is an urban vs. rural difference in these symptoms’ distribution, technology and telehealth interventions can be beneficial and cost-saving to rural populations.

## Figures and Tables

**Table 1:** Nurse-led Symptom Science Studies on Improving Fatigue in PLWH.

Date/Location Authors Design	UCSF Symptom Management Model			Measurements
	Symptom Experience	Symptom Management Strategy	Outcome (Symptom Status)	
2015/US Jaggers JR, Hand GA, et al.RCT	N = 44 PLWH, >18 years old, having no known opportunistic infections and being physically able to complete the exercise intervention. **CON: *n* = 20** **EXP: *n* = 24**	Exercise intervention: - 6-week, 30 min. sessions, twice weekly, moderate intensity of 50–70% of their age-predicted max heart rate - Followed by upper and lower body resistance training for 20 min	⇒ No statistically significant change in fatigue (*p* = 0.175). ⇒ Significant decrease (*p* = 0.003) in self-reported mood disturbance and in total AUC of salivary cortisol (*p* < 0.001).	Profiles of Mood States (POMS) (fatigue subscale); HIV Symptom Distress Scale (SDS); Perceived Stress Scale (PSS); salivary cortisol
2019/US Goulding D, Wilson MP, et al. RCT	N = 69 adults, ages 50–75 years **CON: *n* = 32 PLWH** **EXP: *n* = 37 HIV negative**	Exercise intervention: - − 12 weeks of moderate intensity exercise 3 times per week - Then randomized to complete another 12 weeks of moderate or high intensity exercise	⇒ Higher intensity exercise was associated with significantly improved vitality/fatigue scores (4.1 [0.8, 7.3], *p* = 0.02) ⇒ Week 0–12 change in EXP: −2.9 (−6.3, 0.6), *p* = 0.10. ⇒ Week 13–24 change in EXP: −2.6 (−6.8, 1.7), *p* = 0.23. ⇒ Week 13–24 change in high intensity EXP (ref. moderate): 4.1 (0.8, 7.3), *p* = 0.02 ⇒ No improvement in depression or QOL	Center for Epidemiological Studies-Depression (CES-D); Short Form Health Survey (SF-36)
2016/US Barroso J, Bengtson AM, et al. Secondary analysis from a parent RCT, ‘SLAM DUNC Study’	N = 234 PLWH, ages 18–65, screened positive for depression on the Patient Health Questionnaire-9 (score ≥10), and were confirmed to have current major depressive disorder on the Mini-International Neuropsychiatric Interview (MINI)	Depression treatment model called measurement-based care (MBC): - HIV provider makes final decisions about the treatment plan based on antidepressant treatment recommendation form the clinically supervised depression care manager	⇒ Baseline fatigue intensity score = 6.9 on a 1–10 scale; at 6 months = 5.4; at 12 months = 5.3; Nearly ¾ continued to have severe fatigue at 6 and 12 months ⇒ Partial depression response associated with an average decrease of -0.52 points in fatigue severity (95% CI: −1.10, 0.06); full depression response associated with an average decrease of −2.01 points in fatigue severity (95% CI −2.67, −1.41)	HIV-Related Fatigue Scale (HRFS); Hamilton Depression Rating Scale (HAM-D)
2016/US Doerfler RE and Goodfellow L. RCT	N = 18 PLWH, age > 18, receiving ART, and had 1 or more symptoms that they believed were caused by ART. **CON: *n* = 9** **EXP: *n* = 9**	Cognitive behavioral therapy (CBT) - Delivered by a psychologist - Three, 50-min instructional sessions with progressive muscle relaxation therapy and guided imagery, over a 3-month period, at approximately 2, 4, and 6 weeks.	⇒ Usual fatigue scores in the EXP group (median 42) were significantly lower than in the CON group (median 60) at 2 months, *U* = 12.50, *z* = −2.01; *r* = −0.47, *p* < 0.05 ⇒ Worst fatigue scores in the EXP group (median 59) were significantly lower than in the CON group (median 73) at 2 months, *U* = 15.50, *z* = −1.16, *r* = −0.40, *p* < 0.05 ⇒ At 90 days, intervention group showed no significant difference on usual fatigue and worse fatigue than control group.	Visual Analog Scales (VAS): one each for nausea, pain, anxiety, fatigue, medication adherence (3-day recall); 100 mm in length; Medical Outcomes Study 36-Item Short Form (SF-36); Side Effect Reducing Medication (SERM) use—no meds for fatigue
2018/China Zhu z, Hu Y, et al. Quasi-experimental implementation study	N = 126 PLWH, >18 years old, and self-reported at least one symptom (fever, diarrhea, fatigue, pain, skin lesion, oral mucosal lesion, cognitive disorder, anxiety, depression, and consumptive symptoms) during the past four weeks **CON: *n* = 61** **EXP: *n* = 65**	Tailored intervention based on the symptom management guidelines. - While the intervention was said to be evidence-based and multidisciplinary in implementation, it was not specified.	⇒ Fatigue at Time 1: EXP 41, CON 36, *p* = 0.72; fatigue at Time 2: EXP 27, CON 28, *p* = 0.72; fatigue at Time 3: EXP 24, CON 27, *p* = 0.47 ⇒ Frequency of fatigue was lower in the intervention group but was not statistically significant.	Sign and Symptom Checklist for Persons with HIV disease (SSC-HIV); Center for Epidemiological Studies—Depression (CES-D); Simplified Chinese Version of HIV Adaptation of the Medical Outcomes Questionnaire (MOS-HIV)
2019/US Barroso J, Madisetti M, Mueller M. Feasibility test	N = 25 adult PLWH, reported chronic (at least 3 months’ duration) and moderate to severe fatigue (Score above 5 on the HIV-Related Fatigue Scale)**CON: *n* = 14** **EXP: *n* = 11**	Mobile application ‘mHealth’ intervention: - The app was developed based on Antoni et al.’s Cognitive Behavior Stress Management * (CBSM) intervention - 10 weekly CBSM modules on the app, which was on their cell phones - Module topics were stress-related education, relaxation skills, coping strategies, interpersonal skills, and problem-solving skills	⇒ Participants stated that the intervention was credible, acceptable, and accessible. ⇒ Significant improvements in fatigue intensity (from 64.2 to 59.7) and overall fatigue-related functioning (from 6.6 to 4.2).	HIV-Related Fatigue Scale (HRFS); PROMIS Fatigue Scale—Short Form 6a; State-Trait Anxiety Inventory (STAI)—State Form; Beck Depression Inventory (BDI); Life Experiences Scale (LES); Credibility/Expectancy Questionnaire; Barriers to Treatment Participation Scale
2019/US Lee KL, Jong SS, Gay CL RCT	N = 51 PLWH, >45 years of age, unemployed, retired, or on disability, and had experienced fatigue during the past week. **CON: *n* = 26** **EXP: *n* = 25**	A modified Sleep B.E.T.T.E.R behavioral educational intervention with a tote bag intervention kit (a white noise machine or fan, a night light and eye mask, caffeine-free teas, a lavender sachet, and nasal dilator strips) - Initial one-hour in-person session with sleep technologist - Weekly motivational telephone session for 4 weeks - Monthly assessment over 3 months	⇒ EXP group had significantly lower FSS scores (*p* < 0.05) and fatigue symptom burden over time, particularly in the frequency dimension. ⇒ Secondary outcomes for reducing daytime naps and improving sleep quality were also significant (*p* < 0.05). ⇒ For pre-post comparison, both groups had significantly less fatigue symptom burden (*p* = 0.003) with a significant group x time interaction (*p* = 0.044). ⇒ In the model with 4 time points, both groups still reduced their fatigue burden over time (*p* = 0.006), but the interaction was no longer significant.	Fatigue Severity Scale (FSS); Memorial Symptom Assessment Scale (MSAS); Physical Activity Questionnaire (PAQ); step counts with Fitbit flex; Hospital Anxiety and Depression Scale (HADS); PSQI; Medical Outcomes Survey (MOS) physical and mental function scales, and Sleep Behaviour Rating Scale (SBRS)

Note: **CON**: control group; **EXP**: experimental group; **RCT**: randomized control trial, * Antoni MH, Ironson G, Schneiderman N. (2007). *Cognitive-behavioral stress management for individuals living with HIV: Facilitator guide*. New York: Oxford University Press. Antoni MH, Ironson G, Schneiderman N. (2007). *Cognitive-behavioral stress management workbook*. New York: Oxford University Press.

**Table 2:** Nurse-led Symptom Science Studies on Improving Sleep Disturbances in PLWH.

Date/LocationAuthorsDesign	UCSF Symptom Management Model			Study Measurements
	Symptom Experience	Symptom Management Strategy	Outcome (Symptom Status)	
2001/US Phillips KD, Skelton WD Quasi-experimental	N = 21 PLWH ages between 29–50, with 3 or more sleep disturbances per week and less than 5 PSQI score.	Individualized acupuncture treatment: - Five-week (10 treatments), two evening group sessions per week based on Montakab’s study of the effects of acupuncture on insomnia.	⇒ Significant improve in sleep quality (*p* = 0.01) ⇒ Improve in amount of sleep (*p* = 0.05), minutes awake (*p* = 0.05), sleep percentage (*p* = 0.05), wake percentage (*p* = 0.05), and sleep ratio (*p* = 0.05). ⇒ CSQI improved (*p* = 0.001) ⇒ No significant change in mid-sleep awakenings (*p* = 0.92), minutes awake after mid-sleep awakenings (*p* = 0.15), sleep latency (*p* = 0.87)	PSQI, Wrist Actigraphy, Current Sleep Quality Index (CSQI), Pain Rating Scale
2003/US Dreher HM RCT	N = 88 PLWH (ages 19–64) experiencing insomnia or any other sleeping problem on an occasional or frequent basis with PSQI > 5; taking antiretroviral medication for HIV/AIDS; caffeine intake daily. **CON: *n* = 44** **EXP: *n* = 44**	Withdraw from caffeine using a Gradual Withdrawal from Caffeine Protocol and then avoid all caffeine sources for 30 days. (Instructions for self-management of caffeine-withdrawal headache, record caffeine consumption in 4-week Caffeine Tracking Diary)	⇒ Sleep improved by 35% in subjects who reduced their caffeine intake by 90% or greater from baseline for 30 days versus 6% among control group subjects. ⇒ Post-PSQI was still low after intervention (mean = 7.4, *SD* = 3.3). ⇒ The intervention improved quality of sleep but could not resolve the sleep disruptions caused by myriad of other cofactors.	PSQI, Perceived Well-being Scale-revised, MOS-HIV Health Survey
2008/US Hudson AL, Portillo CJ, Lee KA. Quasi-experimental	Quasi-experimental N = 30 women with HIV (with or without sleep problems)	Sleep B.E.T.T.E.R intervention (bedroom, exercise, tension, time to sleep, eating, rhythm) - One-time 30–45-minute educational session and individualized goal setting for each participant. Education session developed based on sleep hygiene principles.	⇒ Significant improvements in sleep disturbance: (*p*< 0.001), circadian rhythm parameters (*p* < 0.05) and perception of sleep. ⇒ No change in total sleep time at night, sleep onset, number of awakenings or duration of awakening. ⇒ Significant decrease in daytime napping (*p* = 0.02).	PSQI, Wrist Actigraphy, General Sleep Disturbance Scale (GSDS)
2019/US Lee KA, Jong S, Gay CL. RCT	N = 51 PLWH, >45 years of age, unemployed, retired, or on disability, and had experienced fatigue during the past week. **CON: *n* = 26** **EXP: *n* = 25**	A modified Sleep B.E.T.T.E.R intervention with a tote bag intervention kit (a white noise machine or fan, a night light and eye mask, caffeine-free teas, a lavender sachet, and nasal dilator strips) - Initial one-hour in-person session with sleep technologist - Weekly motivational telephone sessions for 4 weeks - Monthly assessment over 3 months	⇒ Significant decrease in FSS, frequency of fatigue (*p* < 0.05) and physical and mental functioning. ⇒ Significant decrease in daytime naps and increase in sleep quality (*p* < 0.05). ⇒ No improvement in sleep medication use, activity, anxiety, or depression.	PSQI; Fatigue Severity Scale (FSS); Memorial Symptom Assessment Scale (MSAS); Physical Activity Questionnaire (PAQ); Hospital Anxiety and Depression Scale (HADS); Medical Outcomes Survey (MOS) physical and mental function scales, and Sleep Behaviour Rating Scale (SBRS); step counts with Fitbit flex
2013/US Webel AR, Moore SM, Hanson JE, Patel SR, Schmotzer B, Salata RA Feasibility test	N = 40 living with HIV, between 45 and 75 years old.	SystemCHANGE^TM^ -HIV intervention: - 10 weekly sessions on different topics of HIV management, including sleep hygiene and behavioral modification strategies, delivered by a health educator or registered nurse	⇒ Participants found the intervention useful (77%), worth the time (76%), and useful for the future (94%). ⇒ Non-significant improvements in sleep-related measurements.	Wrist actigraphy; PROMIS Sleep Disturbance and Sleep-Related Impairments Scales; HIV/AIDS Targeted QOL
2018/US Buchanan DT, McCurry SM, Eilers K, Applin S, Williams ET, Voss JG. Feasibility test	N= 22 PLWH who had difficulty with sleep onset, waking after sleep onset, or awakening prior to intended rise time, ≥ 3 nights per week, lasting at least 1 month, and associated with a daytime consequence. **CON: *n* = 10** **EXP: *n* = 12**	Brief Behavioral Treatment for Insomnia (BBTI): Four weekly sessions - session 1, 3 in person - session 2, 4 by telephone	⇒ Acceptability: favorable ⇒ Significant improvement on sleep measures (ISI, PROMIS-Sleep, SHPS, and SDQ) with a large effect size (1.11–1.91). ⇒ No difference in PHQ-8 or PROMIS-fatigue. ⇒ Common sleep hygiene problem: variable bedtimes and rise times, watching television, or consuming caffeine.	PSQI; Insomnia Severity Index (ISI); Sleep diary; PROMIS-sleep and fatigue; Sleep Hygiene Practice Scale (SHPS); Sleep Disturbance Questionnaire (SDQ); Patient Health Questionnaire-8(PHQ-8); Feasibility measurement
2019/US Cody SL, Fazeli PL, Crowe M, et al. RCT	N = 66 adults >50 years old **Arm 1:** HIV-positive tDCS + SOP training **Arm 2:** HIV-positive sham tDCS + SOP training **Arm 3:** HIV-negative tDCS + SOP training **Arm 4:** HIV-negative sham tDCS + SOP training	10 one-hour sessions of speed of processing (SOP) with transcranial direct current stimulation (tDCS) over 5 weeks	⇒ tDCS with SOP training did not improve sleep. ⇒ Significant improvement in LPCT for the HIV-positive tDCS with SOP training group. ⇒ Significant improvement on DSSC for both the HIV-positive and HIV-negative sham tDCS with SOP training groups. ⇒ UFOV improved in all groups.	PSQI; Letter and Pattern Comparison Test (LPCT); Wechsler Adult Intelligence Scale (WAIS); Digit Symbol Substitution and Copy Tests (DSSC); Useful Field of View (UFOV)

Notes: **CON**: control group; **EXP**: experimental group; **PSQI:** Pittsburgh Sleep Quality Index; **RCT:** randomized control trial.

## Data Availability

All the data are available in the manuscript.
